# Relugolix: A new kid on the block among gonadotrophin-releasing hormone antagonists

**DOI:** 10.1080/2090598X.2021.1994231

**Published:** 2021-10-24

**Authors:** Charalampos Fragkoulis, Ioannis Glykas, Athanasios Dellis, Iraklis Mitsogiannis, Athanasios Papatsoris

**Affiliations:** a Department of Urology, General Hospital of Athens ‘G. Gennimatas’, Mesogeion Avenue 154, Athens, P.C, 115 27, Greece; b Second Department of Surgery, Aretaieion Hospital,School of Medicine, National and Kapodistrian University of Athens, Athens, Greece; c Second Department of Urology, Sismanoglio Hospital, School of Medicine, National and Kapodistrian University of Athens, Athens, Greece

**Keywords:** Relugolix, novel drugs, androgen-deprivation therapy, prostate cancer

## Abstract

Androgen-deprivation therapy (ADT) is the cornerstone of metastatic prostate cancer treatment. ADT can be achieved through surgical castration, or it may be induced either by gonadotrophin-releasing hormone (GnRH) agonists or GnRH antagonists. GnRH antagonists provide a more rapid castration alongside with a safer profile regarding adverse events. Degarelix is the sole GnRH antagonist used in clinical practice. Injection site reactions are the commonest adverse events related to the use of degarelix. Relugolix, a novel molecule, represents the first orally administered United States Food and Drug Administration approved GnRH antagonist, with clinical efficacy equal to that of the established ADT regimens. The main advantages of relugolix are the avoidance of the injection site reactions of GnRH antagonists such as degarelix alongside its patient-friendly oral administration. The aim of the present review article is to present novel data regarding the role of relugolix as ADT for the treatment of prostate cancer.

**Abbreviations**: ADT: androgen-deprivation therapy; FDA: United States Food and Drug Administration

## Introduction

Androgen-deprivation therapy (ADT) is nowadays the cornerstone of metastatic prostate cancer treatment. Patients advancing into castration resistant status should also remain under continuous ADT regardless of other treatment modalities applied. Furthermore, patients diagnosed with localised or locally advanced prostate cancer scheduled for radiotherapy are also submitted to ADT with variable duration based on risk stratification [1]. ADT can be achieved through surgical castration, or it may be induced either by GnRH agonists or antagonists [2].

GnRH antagonists provide a more rapid castration compared with GnRH agonists as they bind to the pituitary GnRH receptors in a competitive way resulting in testosterone suppression to castration levels avoiding at the same time the testosterone ‘flare-up’ phenomenon described when GnRH agonists are used [3]. As GnRH antagonists do not provoke a testosterone flare-up phenomenon and that testosterone surges during maintenance with GnRH agonists are also avoided, they present an excellent treatment option in patients with symptomatic prostate cancer, especially those who present with bone pain, ureteric or BOO, bone fractures or spinal cord compression [4]. In addition, simultaneous administration of antiandrogen is not necessary. Despite initial drawbacks related to histamine mediated adverse events described with abarelix use, degarelix is currently widely used as the sole GnRH antagonist approved both in Europe and the USA [4]. Degarelix is a third generation GnRH antagonist administered by monthly injections starting at 240 mg the first month followed by 80 mg monthly [5]. As all available options regarding ADT are based on injectable formulas, there is wide interest in developing orally administered GnRH antagonists in order to avoid severe injection side reactions including pain, erythema, pruritus and swelling, which are common with degarelix [6].

In 2020, the United States Food and Drug Administration (FDA) approved the first orally administered GnRH antagonist, relugolix (TAK-385) based on the results of the HERO trial (ClinicalTrials.gov Identifier: NCT03085095) [7]. The aim of the present review is to present all novel data regarding the safety and efficacy of relugolix as the first orally administered FDA-approved GnRH antagonist for the treatment of prostate cancer.

## Relugolix development

Based on previous studies aiming to develop GnRH antagonist molecules, Miwa et al. [8] managed to synthesise a highly potent, orally administered GnRH antagonist named TAK-385, with the chemical name: 1-{4-[1-(2,6-difluorobenzyl)-5-[(dimethylamino)methyl]-3-(6-methoxypyridazin-3-yl)-2,4-dioxo-1,2,3,4-tetrahydrothieno[2,3-d] pyrimidin-6-yl] phenyl}-3-methoxyurea, and the advantage of this molecule is that it presents superior *in vivo* GnRH antagonistic characteristics, as well as a reduced inhibition of cytochrome P450 compared to other molecules such as sufugolix. In animal models, TAK-385 (relugolix) was orally administered and resulted in continuous but reversible suppression of the hypothalamic–pituitary–gonadal axis, thus emerging as a possible therapeutic option in hormone-dependent conditions such as endometriosis, uterine fibrosis, and prostate cancer [9].

## Pharmacokinetics and pharmacodynamics

Information regarding pharmacokinetic and pharmacodynamic characteristics of relugolix were available after a three-part, randomised, double-blind, placebo-controlled, dose-escalating, phase I trial involving 176 healthy males. Regardless of dosage, relugolix was well absorbed with a median time to maximum plasma concentration (T_max_) of 1.01–1.59 h. Within the dosage range of 20–180 mg/day, elimination half-life was measured around 36–65 h. The maximum plasma concentration was documented at 180 mg/day. Drug concentration is severely affected by food intake, while its urine excretion is very low. Relugolix doses of ≥80 mg/day achieved sustainable testosterone suppression (testosterone castration levels <50 ng/dL). After treatment cessation testosterone levels returned to normal in most individuals in <28 days [10].

## Clinical efficacy

Clinical efficacy of GnRH antagonists is well established. Klotz et al. [11] evaluated degarelix in an open-label randomised phase III trial in terms of both efficacy and safety and compared with leuprolide, degarelix proved not to be inferior in achieving and maintaining testosterone suppression for a treatment period of 12 months. A major benefit of its use was the rapid testosterone suppression without a testosterone flare phenomenon plus a lower risk of PSA progression in patients with PSA level of >20 ng/dL. Patients participating in the aforementioned trial were offered the possibility of entering a 5-year extension period. Patients receiving degarelix continued their treatment in the same way and patients receiving leuprolide were switched to either degarelix 240/80 mg or degarelix 240/160 mg with all patients converting to degarelix 80 mg after regulatory approval. In terms of results, testosterone and PSA suppression were similar with the pivotal study. Patients who were constantly under degarelix presented a progression-free survival (PFS) hazard rate consistent with the extension phase of the trial [12]. Based on the results of a pooled analysis performed by Klotz et al [13] extracting data from five prospective phase III/IIIb trials, degarelix presented improved PSA reduction, longer PFS, as well as longer overall survival compared with GnRH agonists.

For relugolix, Suzuki et al. [14] performed an open-label phase I trial in order to estimate the optimal dose scheme. TAK-385 was administered in Japanese patients diagnosed with non-metastatic prostate cancer. In the dose escalation part of the trial, TAK-385 was administered at 320 mg as a loading dose on day 1 followed by 80 mg daily thereafter or at doses of 320/120, 320/160, or 360/120 mg for a total period of 28 days. Part two of the trial was based on randomisation of patients either to receive TAK-386 at 320/80 mg or 320/120 mg for up to 96 weeks. In terms of results, TAK-385 was absorbed in a similar way regardless of the dosage and all schemes resulted in testosterone suppression to castration levels during the first week of treatment. Although no dose-limiting toxicities were documented, the optimal dose for clinical effect was 320/80 mg.

The pivotal phase III study of relugolix that led to FDA approval was published recently by Shore et al. [7]. The investigators randomly stratified patients diagnosed with advanced prostate cancer either to receive relugolix or leuprolide for a treatment period of 48 weeks. Relugolix was administered orally at a single loading dose of 320 mg followed by 120 mg daily. While leuprolide was administered by injection every 3 months. The study’s primary end-point was the achievement of a sustained testosterone suppression to castrate levels during the treatment period. Secondary end-points such as non-inferiority regarding the primary end-point, testosterone levels on day 4 and castration levels set at <20 ng/dL on day 15 were also evaluated. Relugolix managed to suppress testosterone and maintain castration levels in a superior way compared with leuprolide. Apart from superiority regarding the primary end-point, relugolix demonstrated superiority over leuprolide in all secondary end-points as well. In a subgroup of 184 patients, testosterone recovery evaluation was possible. The mean testosterone levels 3 months after treatment cessation were 288.4 ng in the relugolix arm and 58.6 ng in the leuprolide arm. Furthermore, relugolix was safer in terms of risk of major cardiovascular events presenting a 54% lower risk.

Relugolix was also tested in the neoadjuvant and adjuvant setting in combination with external beam radiotherapy in patients with intermediate-risk prostate cancer eligible for 6 months of ADT. The main objective of the phase II trial performed by Dearnaley et al. [15] was to investigate whether relugolix produces rapid and durable testosterone castration. Secondary end-points included testosterone kinetics, PSA levels, prostate volume, quality of life, and safety profile as well. Patients were randomised either to receive relugolix *per os* 320 mg on day 1 followed by 120 mg daily or degarelix injection formula monthly for a total period of 24 weeks. Both drugs achieved excellent castration results but when lower testosterone castration levels were applied (<20 ng/dL) relugolix achieved that goal in 82% of patients while degarelix in 68%. The median time to castration was 3 days in the degarelix arm and 4 days in the relugolix arm. Effects on PSA levels and prostate volume were similar among the two groups. Regarding testosterone recovery, 52% of patients who received relugolix presented normal testosterone levels 3 months after treatment discontinuation compared with 16% of patients who received degarelix. No difference was noted in adverse events with the most common being nausea in both groups.

## Safety

Safety of GnRH antagonists is well documented by large phase III trials in which degarelix presented a similar safety profile as GnRH agonists [13,16]. Degarelix presents injection site reactions such as pain, erythema, pruritus and swelling more frequently than GnRH agonists. In terms of hot flashes, erectile dysfunction, decreased libido and renal-related adverse events, no difference was recorded comparing degarelix with GnRH agonists [13]. Recently, Abufaraj et al. [16] published the results of a meta-analysis regarding the clinical safety and oncological outcomes of GnRH agonists vs antagonists. The authors concluded that GnRH antagonists were associated with more injection site reactions in a statistically significant way. There is a trend of less serious adverse events among patients under GnRH antagonist treatment (9.8% vs 11%). Both groups presented similar dropout rates due to adverse events. Furthermore, GnRH antagonist proved to be safer in terms of musculoskeletal and cardiovascular adverse events.

Comparison between GnRH agonists and antagonists in terms of therapy-related cardiovascular risk represents a major field of interest regarding safety of ADT [16]. Patients with prostate cancer may also present several cardiovascular comorbidities at the time of prostate cancer diagnosis [16]. In such a fragile group of patients, GnRH antagonists may be preferred to GnRH agonists, as their use is correlated with a statistically significant lower risk of cardiac events during treatment [17]. Relugolix appears to have a safe drug profile regarding adverse events. The documented side-effects were all mild, with the most prevalent being bradycardia, headache, and hot flushes [10]. Relugolix is a novel drug and therefore all information regarding safety is drawn from pivotal studies as no real-life data are yet available. In the HERO trial, which eventually led to FDA approval, the most common adverse event related with relugolix use was the hot flashes (54.3%). In terms of severe adverse events, fatal events were reported in 1.1% of patients under relugolix therapy compared to 2.9% of patients under leuprolide therapy. Following the pattern of GnRH antagonists, relugolix also reduced the incidence of major cardiovascular events by 54% compared with leuprolide, especially in patients with cardiovascular comorbidities. On the other hand, relugolix patients more frequently reported diarrhoea than the leuprolide patients (12.2% vs 6.8%) but all cases were of mild or moderate severity, thus no drug discontinuation was reported for this adverse event [15].

As data are still limited, the only systematic review focussing on cardiovascular risk including patients who received relugolix is the one published very recently by Cirne et al. [18]. The Authors conclude that regarding cardiovascular adverse events when we compare GnRH antagonists with GnRH agonists, GnRH antagonists prove to be safer. Patients who received relugolix were included in this review. These results are underpowered by several study design limitations with most important being the short duration of follow-up.

## Commentary

A major concern regarding the use of relugolix in everyday clinical practice is the patient adherence to treatment. Although in the HERO study compliance was extremely high (99%), this may not reflect the real-life setting. Data support that food intake decreases the drug absorption by 50%, thus it should be administered on an empty stomach. Furthermore, plasma protein binding is ~70%, which may result in interactions with several inducers or inhibitors of p-glycoprotein. On the other hand, relugolix is an orally administered GnRH antagonist and that makes it a more patient friendly medicine compared with injectable depot formulae, as local site reactions are quite common in these kinds of formulae. The most important asset of relugolix over degarelix in order to encourage patient adherence is the fact that patients avoid the injection site reactions related with degarelix. Moreover, relugolix provides a more flexible dosing profile and is ideal for prompt cessation because of adverse events or intolerance. Relugolix acts as a pure GnRH antagonist, thus the concurrent administration of antiandrogen is avoided as no flare phenomenon is present.

To conclude, data support that the novel GnRH antagonist relugolix provides the first orally delivered GnRH antagonist option. It presents excellent testosterone suppression to castration levels and has less cardiovascular events compared to leuprolide. Relugolix is delivered orally, in a more patient friendly way, providing the advantage of avoiding injection depot formulas, which are related with frequent adverse events in the local site of injection.
